# Antigen detection of Talaromyces infection in the US: Case report

**DOI:** 10.1016/j.mmcr.2026.100778

**Published:** 2026-03-06

**Authors:** Emily E. Evans, Jennifer Spicer, Vince Marconi, Thuy Le

**Affiliations:** a Emory University, 100 Woodruff Circle, Atlanta, GA, 30321, United States of America; b Division of Infectious Diseases and International Health, Duke University School of Medicine, Durham, NC, 27708, United States of America; c Tropical Medicine Research Center for Talaromycosis, Pham Ngoc Thach University of Medicine, Ho Chi Minh City, Viet Nam

**Keywords:** Talaromycosis, *Talaromyces marneffei*, Antigen testing, Mp1p antigen

## Abstract

Talaromycosis is an invasive fungal infection endemic in Southeast Asia and has emerged as a leading cause of HIV-related death in the highly endemic countries of Vietnam, Thailand and China. Travel-related cases have been increasingly reported in immunocompromised people since the 1980s. Lack of clinical suspicion and non-culture-based diagnostics prevent timely, accurate diagnosis and treatment. Here we describe a travel-related case of talaromycosis in whom the diagnosis was confirmed using Mp1p antigen detection, highlighting its clinical utility.

## 1 Introduction

Talaromycosis is an invasive fungal infection caused by the environmental dimorphic fungus endemic in Southeast Asia *Talaromyces marneffei* (TM*)*. Talaromycosis disproportionately affects people who are immunocompromised, particularly people with advanced HIV disease (AHD) having a CD4^+^ T-cell count of <100 cells/μl, in whom talaromycosis accounts for 4 to 20% of AHD-related hospital admissions and is a leading cause of death in the hyperendemic countries of Vietnam, Thailand, and China [1]. Despite appropriate antifungal therapy, mortality is up to 30% in people with AHD, and 50% in people without HIV, largely due to lack of disease awareness resulting in delayed diagnosis [2]. Despite being a major cause of AHD-related deaths, talaromycosis has been neglected by global and regional HIV responses [1]. Diagnosis and treatment options remain severely limited, prompting the inclusion of talaromycosis in the World Health Organization priority fungal pathogen list in 2022 to promote further research and public health attention [3].

While highly endemic in Southeast Asia, travel-related cases have been reported worldwide, with an incubation period ranging from a few weeks to many years (latent infection) after exposure [4]. Given the low frequency of travel-related cases and high mortality with late diagnosis, clinical suspicion is key to making a timely diagnosis, allowing early initiation of antifungal therapy. The clinical presentation of talaromycosis is non-specific and diverse, ranging from clinically silent infection in immunocompetent hosts, to upper and lower respiratory infections in mildly immunosuppressed individuals, to disseminated disease involving the upper and lower respiratory tracts, gastrointestinal lesions, skin lesions, hepatosplenomegaly, lymphadenopathy, blood, skin, and bone marrow invasion in people with profound immunosuppression.

Even with high clinical suspicion and disseminated disease, diagnosis remains challenging. Blood culture is the mainstay of diagnosis but does not detect infection during the early stage of disease, lacks sensitivity (50-70%), and requires waiting between 5 and 28 days for positive results [5]. Diagnosis and treatment of talaromycosis are further delayed in non-endemic regions due to lack of mycology expertise and absence of non-culture-based diagnostic tools. Delays in diagnosis are associated with worse clinical outcomes and an increase in mortality [2]. We describe a challenging case of culture-negative talaromycosis in a returning traveler from Southeast Asia to the United States who had multiple hospital admissions over seven months without a diagnosis until clinical suspicion prompted antigen testing. The diagnosis was confirmed by antigen positivity in both serum and urine samples, and evidence of clinical, mycological, and radiographical resolution on antifungal therapy. We review current data on the diagnostic performance and clinical utility of antigen testing for the diagnosis of talaromycosis.

## 2 Case

**Fig. 1:**
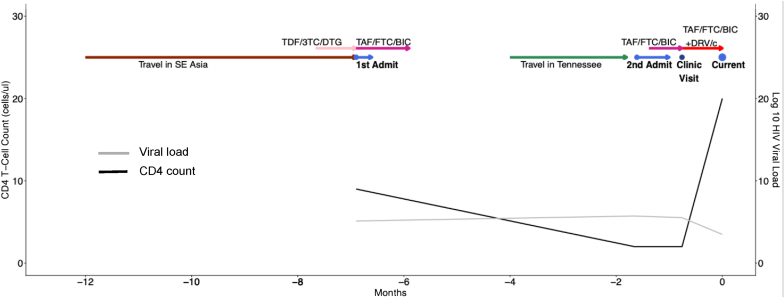
Clinical timeline from most recent exposure to admission where ultimate diagnosis was confirmed. Additionally included are start/stop dates for ART and associated viral load and CD4 data.

A 33-year-old man with a past medical history of HIV diagnosed in 2015 presented to an urban hospital in Georgia with fevers and shortness of breath. The timeline of patient presentation and diagnosis is shown in Fig. 1.

*First admission:* The patient traveled in Hong Kong, Bangkok, Ho Chi Minh City, and throughout Malaysia for 6 months spending his time visiting friends and family in urban centers. Upon returning to the US, he presented directly to the hospital (Day −212) with dizziness, generalized weakness, and dark loose stools. He was found to be anemic with a hemoglobin of 5.6 mg/dl. He was transfused with two units of packed red blood cells and underwent esophagogastroduodenoscopy and colonoscopy with evidence of gastritis. Pathology from a duodenal biopsy revealed focal nonspecific intraepithelial lymphocytosis. Fungal stains were not performed. He was discharged with a diagnosis of parvovirus B19 infection (positive parvovirus B19 PCR in blood). He had started antiretroviral therapy (ART) three weeks prior to admission (Day −233) with tenofovir disoproxil fumarate, lamivudine, and efavirenz while in Vietnam. During admission, his CD4^+^ T-cell count was 9 cells/μl (with 2% CD4/total lymphocyte count) and HIV-1 plasma RNA viral load was 127,396 copies/ml (log 5.11). He was transitioned to bictegravir/emtricitabine/tenofovir alafenamide during admission. Despite multiple attempts to coordinate a clinic appointment, he was lost to follow up.

**Fig. 2:**
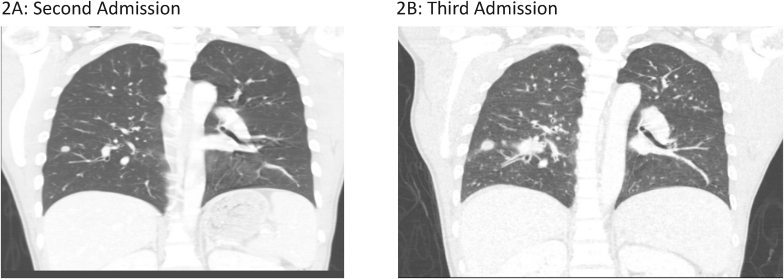
Computed tomography (CT) chest of patient during his second (Fig. 2A) and third (Fig. 2B), respectively showing enlarging lung nodules and new miliary appearance.

*Second admission:* Five months following discharge, the patient re-presented (Day −49) with two-months of fevers, chills, weight loss, shortness of breath, and was found to have a hemoglobin of 5.4 mg/dl (reticulocyte index 0.01), CD4 count of 2 cells/μl (0%), and a 9 mm pulmonary nodule in his right lower lobe on chest computed tomography (CT) scan (Fig. 2A). He had a transbronchial lung biopsy with culture that grew *Streptococcus anginosis*, *Staphylococcus aureus*, and *Actinomyces spp*. Pathology of the nodule was notable for clusters of macrophage and lymphoid cells with enlarged nuclei without clear granulomas, however, fungal stains were not performed. Additional findings included fibrosed stroma with fragments of ischemic or necrotic stroma. Cytology did not show any fungal organisms. Evaluation for tuberculosis and fungal infections included sputum smears and cultures for fungi, acid-fast bacilli (AFB) and a tuberculosis PCR were negative. His urine *Histoplasma* antigen by MiraVista enzyme immunoassay (EIA) was positive at 0.3 ng/ml (positive cut off 0.2 ng/ml). Serum quantitative parvovirus 19 PCR was positive at > 8 log copies/ml. He received blood transfusions, 2 doses of IVIG for parvovirus B19 infection, and 2 weeks of IV ceftriaxone followed by 3 weeks of amoxicillin for his pulmonary infection, presumably caused by *Actinomyces spp*. Given low-level *Histoplasma* antigen positivity, negative fungal culture, *Actinomyces spp.* isolated on bacterial culture, and symptom improvement, he was not treated with antifungal therapy. His HIV RNA during the second admission was 522,810 copies/ml (5.72 log). He restarted bictegravir/tenofovir alafenamide fumarate/emtricitabine (Day −42) with follow-up scheduled in a HIV clinic two weeks after discharge. At his HIV follow-up appointment 4 weeks after ART re-initiation (Day −23), his repeat HIV VL was 328,333 (5.52 log) copies/mL. Given concern for possible integrase resistance, an integrase genotype was obtained, and darunavir and cobicistat were added to his HIV regimen while awaiting results.

**Fig. 3:**
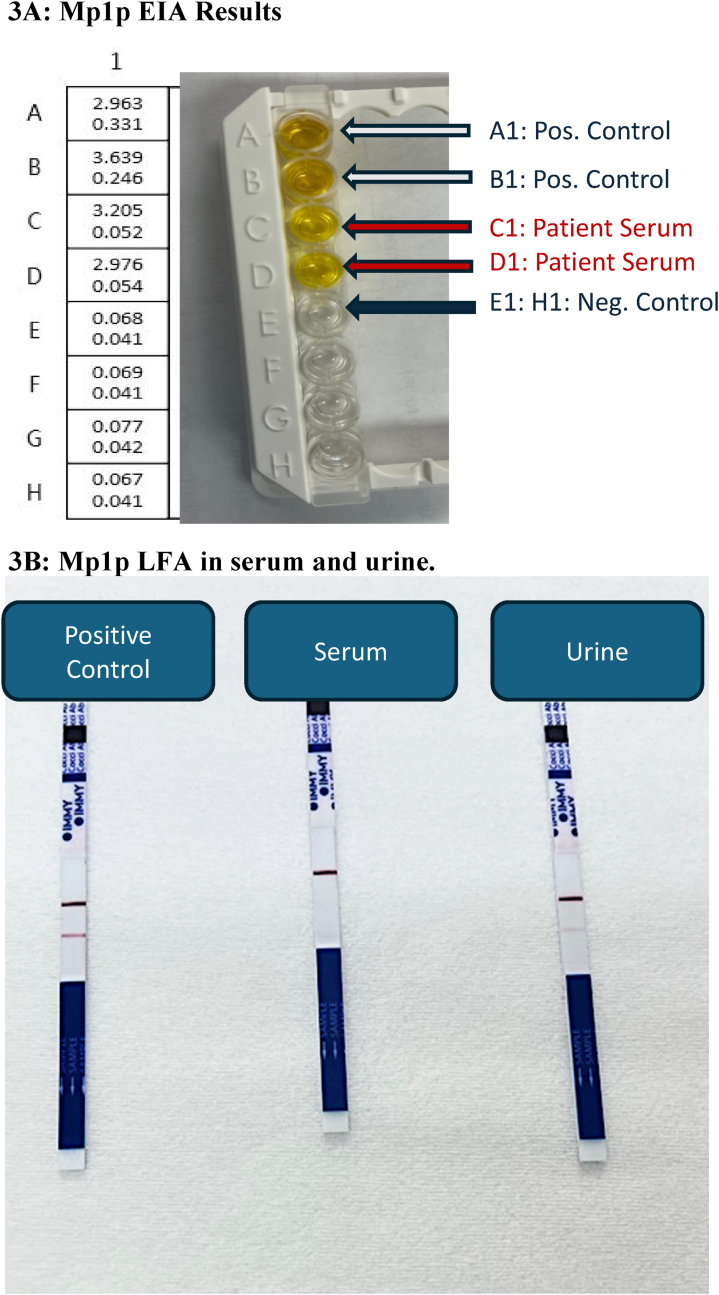
Optical density results from the Mp1p EIA testing in our patient serum are shown in 3A. Wells A and B with positive control demonstrating positive colorimetric change and associated optical density values of 2.963 and 3.639. Wells C and D with patient serum also overtly positive with colorimetric change and associated optical density values of 3.205 and 2.976. Well E is the negative control with optical density of 0.068. LFA results from serum and urine are shown in 3B.

*Third admission*: The patient re-presented three weeks later (Day 0) with fevers, fatigue, shortness of breath, and a 10-pound weight loss. On this admission he was found to have miliary nodules, mediastinal adenopathy, and increasing size of the previous lung nodule on chest CT (Fig. 2B). His HIV VL was 2990 (3.48 log) copies/mL and CD4 count was 20 cells/μl (5%). Fungal sputum culture grew *Candida albicans* which was considered a contaminant. His urine *Histoplasma* antigen level increased to 0.79 ng/ml. Given his recent travel history to Southeast Asia and low-level positivity for *Histoplasma* antigen with known cross-reactivity to other mycoses*,* talaromycosis was suspected [6]. We sent his serum to Dr. Thuy Le's laboratory at Duke University for TM Mp1p EIA testing which was highly positive with an optical density of 3.205 and 2.976 (maximum 4.0) (Fig. 3A). Serum and urine testing by a Mp1p lateral flow assay (LFA) (by Immuno Mycologics, Inc. (IMMY) (Norman, OK) currently undergoing prospective clinical validation, were also positive (Fig. 3B). The diagnosis of probable talaromycosis was made based on a compatible clinical syndrome, an epidemiological risk factor, and positive Mp1p antigen testing in both serum and urine. He was started on 4mg/kg intravenous liposomal amphotericin for 14 days, with rapid clinical improvement, and ultimately discharged on 400mg oral itraconazole for consolidation therapy. Repeat Mp1p antigen testing by LFA in both serum and urine was negative after four months of antifungal therapy and ART.

## 3 Discussion

We present a case of culture-negative antigen-positive talaromycosis in a returning traveler with AHD. In our patient, the rapid and accurate results of antigen testing led to targeted therapy and provided a biomarker for monitoring response to treatment. Antigen diagnosis in this case not only permitted timely and accurate treatment, but also prevented unnecessary infection precautions related to tuberculosis and an invasive procedure to obtain lung biopsy to evaluate for other etiologies.

The European Confederation of Medical Mycology (ECMM) defines probable infection with an endemic mycosis as 1) an epidemiologic exposure, 2) a compatible clinical syndrome, and 3) positive antigen testing (*Histoplasma spp* and *Blastomyces spp*) or antibody testing (*Coccidioides spp*) [7]. Our patient was diagnosed with talaromycosis given his travel history, compatible syndrome, positive Mp1p antigen, clinical and radiographic improvement, and subsequent antigen clearance on antifungal therapy. This patient's prolonged course highlights multiple missed opportunities for diagnosis where a non-culture-based diagnostic could have been pivotal. This patient had multiple AFB blood cultures over this course, but neither fungal blood cultures nor fungal stains on biopsy specimens were ever obtained, underscoring the lack of clinical suspicion in a non-endemic setting. His initial presentation of gastrointestinal bleeding prompted endoscopic visualization revealing inflammatory findings on histology which could be consistent with gastrointestinal talaromycosis, a manifestation seen in 31% of talaromycosis patients [8].

During the second admission, the patient underwent extensive workup for his lung nodule including bronchoscopy alveolar lavage with biopsy as well as AFB and fungal cultures which were unrevealing. Pulmonary manifestations of talaromycosis occur in 50-70% of cases and can have various manifestations including pneumonitis, pulmonary nodules, lobar consolidations, ulcerative lesions and pleural effusions [9]. Only two-thirds of patients with pulmonary talaromycosis have a positive culture from a respiratory specimen and delay in diagnosis incurs an increased risk of death [9]. Timely diagnosis via antigen testing earlier in his course could have led to more rapid initiation of appropriate antifungal therapy. Antigen testing has not been evaluated using respiratory samples, but this should be considered in the future.

Prior to Mp1p results, this patient remained on airborne precautions for possible tuberculosis despite negative AFB sputum smears and *M. tuberculosis* PCR given lack of an alternative diagnosis. Mp1p antigen detection allowed for rapid initiation of amphotericin B, discontinuation of unnecessary precautions with a negative pressure room and respiratory isolation, and prevented the need for a repeated, invasive lung biopsy. This case illustrates the clinical utility of antigen testing on making an earlier diagnosis, improving patient management, avoiding the need for invasive biopsy procedures, and reduction of healthcare utilization costs.

The ECMM recommends rapid diagnosis of talaromycosis by culture or compatible clinical syndrome with characteristic yeast seen on microscopy or histology [10]. However, fungal-specific stains and experienced technicians are required. There have been significant advancements in the development of non-culture-based diagnostics for talaromycosis (Table 1) [11]. Antigen testing using monoclonal antibody (MAb)-based EIA and lateral flow assays (LFA) are in late stages of development and target Mp1p, a fungal cell wall mannoprotein specific to TM, and the MAb-4D1-GNA, targeting a nonspecific antigen in the whole-cell yeast extract of TM. The MAb-Mp1p EIA revealed superior sensitivity and specificity compared to blood culture (84%–86% vs 67%–73%) and could detect infection as early as 16 weeks before growth occurs in culture [12,13]. There was no analytical cross-reactivity with other 11 common clinical fungal pathogens for the Mp1p EIA during assay development nor in clinical validation in patient cohorts [12]. False negative results occur in approximately 10% so a negative test does not rule out talaromycosis. Further research is needed to determine the effect of preceding antifungal therapy on the diagnostic performance of the Mp1p antigen test and their role of screening asymptomatic individuals. The D41 EIA had excellent clinical performance in culture positive cases (sensitivity 89-100%, specificity 100%) but has not been evaluated in patient cohorts. The Mp1p LFA had a sensitivity of 93% and specificity of 99% in a case-control study, and prospective clinical validation for the Mp1p LFA is ongoing [14].

**Table 1:** Diagnostics for talaromycosis.

Investigational *Talaromyces marneffei* test [11]	Sensitivity	Specificity	Population
Mp1p EIA	72-98%	96-98%	Both retrospective and prospective cohorts of patients with AHD
Mp1p LFA	93%	99%	One retrospective cohort in AHD
D41 EIA	89-100%	99-100%	Only retrospective, culture positive cases
Mp1p, D4	92%	100%	One retrospective cohort in AHD
PCR 5.8s rRNA	60-88%	97%	Multiple retrospective cohorts in AHD
PCR 18S rRNA	67%	NE	One retrospective cohort in AHD
PCR ITS region of rRNA	67-77%	NE	One retrospective cohort in culture proven talaromycosis
PCR MP1	70%	100%	One retrospective cohort in AHD an culture proven talaromycosis
aMetagenomic next generation sequencing	97-100%	99-100%	Multiple retrospective cohorts in patients with and without AHD

∗NE = not evaluated.

a Commercially available in the US.

Modern molecular methods of diagnosis utilize quantitative polymerase chain reaction (qPCR) (including TaqMan real-time and loop-mediated isothermal amplification (LAMP)) and metagenomic next generation sequencing (mNGS). qPCR methods target the 18S, 5.8S or internal transcribed spacer (ITS) region of ribosomal RNA or the *MP1* gene. A recent qPCR test targeting 5.8S rRNA was highly sensitive (99%) in blood culture positive cases and could additionally identify 55-70% of blood culture negative cases [15]. mNGS has the benefit of evaluating multiple pathogens simultaneously and was sensitive in retrospective analyses (97-100% sensitive and specific); however, cost and lack of expertise remain a barrier for implementation in most endemic regions [16]. An assay using LAMP in conjunction with LFA may offer a resource-efficient molecular method; however, it has only been studied in one retrospective cohort where it demonstrated 100% sensitivity and specificity [17]. While performance of these molecular methods is promising, they remain expensive, labor intensive, and require high-quality DNA, which can be challenging to obtain in clinical specimens. However, due to very high specificity and positive predictive value, both a positive qPCR or Mp1p antigen test should prompt empiric therapy [10,11].

Cross-reactivity of *Histoplasma* antigen tests with TM is a diagnostic concern, but management for both histoplasmosis and talaromycosis is similar. Commercially available antigen tests for histoplasmosis in the United States include a polyclonal antibody-based EIA and LFA by MiraVista Diagnostics (MVista) (Indianapolis, IN), and a monoclonal antibody-based assay by IMMY (Table 2). Previous studies revealed cross-reactivity of the third generation polyclonal antibody-based MVista *Histoplasma* polysaccharide antigen EIA with other endemic mycoses, including a positive *Histoplasma* antigen test in 4/5 participants with talaromycosis [6]. The MVista *Histoplasma* LFA similarly showed cross-reactivity in 39/52 participants with talaromycosis [18]. A monoclonal antibody-based IMMY *Histoplasma* EIA showed 95% sensitivity and 97% specificity when compared to culture-confirmed histoplasmosis in people with AHD from a multicenter validation cohort in Colombia and Guatemala, regions that are not endemic for TM [19]. Our patient also had epidemiologic exposure to histoplasmosis, hence we cannot exclude the possibility of histoplasmosis co-infection. However, talaromycosis is much more common in Southeast Asia than histoplasmosis. Urine histoplasma antigen levels in people with AHD are generally substantially higher than as observed in our patient [20]. Given the known cross-reactivity of the MVista Histoplasma antigen test with TM, we believe that the weakly-positive urine *Histoplasma* antigen from the MVista polyclonal antibody-based EIA test likely represents a cross-reactivity with TM rather than histoplasmosis co-infection.

**Table 2:** Diagnostic performance of histoplasma antigen.

Histoplasma antigen tests for progressive disseminated histoplasmosis [7]	Sensitivity	Specificity	Cross reactivity with talaromycosis
3rd generation MVista polyclonal Ag EIA	95%	99%	4/5 patients with talaromycosis had a positive test
a4th generation MVista polyclonal Ag EIA	85-95%	99%	Specificity excludes blastomycosis, not specifically tested against talaromycosis
MVista LFA ([18])	78-96%	90-99%	39/52 patients with talaromycosis had a positive test
IMMY polyclonal ALPHA EIA	30-100%	79-99%	Specificity not tested in patients with talaromycosis
IMMY monoclonal GM ASR	76-91%	96-100%	Specificity not tested in patients with talaromycosis
aIMMY monoclonal GM EIA [19]	72-98%	97-98%	Specificity not tested in patients with talaromycosis

∗GM = galactomannan, ASR = analyte specific reagents.

a Commercially available in the US.

Our patient presented during the 3rd hospital admission with miliary nodules in the setting of recent ART initiation. Although his enlarging lung nodule and development of miliary disease without antifungal therapy could be consistent with disease progression, his clinical exacerbation and radiographic evidence of worsening lung disease in the setting of recent initiation of effective ART (demonstrating a 2.24 log decline in HIV viral load) raises the possibility of immune reconstitution inflammatory syndrome (IRIS). IRIS has been described in talaromycosis both as an unmasking and paradoxical phenomenon [21]. In either case, the patient was effectively managed with antifungal therapy and continuation of ART without the need for anti-inflammatory therapy.

## 4 Conclusion

This case illustrates the challenges of diagnosing culture-negative talaromycosis in patients from endemic regions. Mp1p antigen testing offers rapid and accurate non-invasive non-culture-based diagnosis that allows for early treatment initiation and avoids invasive diagnostic procedures. An Mp1p LFA is being developed in the US, which would allow for rapid point-of-care diagnosis of disease, and potentially for screening high-risk immunocompromised individuals, such as those with AHD, before symptom onset. Both Mp1p EIA and LFA are currently limited to research use and the challenges of sending samples to a reference laboratory and to obtain regulatory approval in a non-endemic setting may prove difficult preventing widespread clinical use. Further research is needed to understand the natural history of talaromycosis disease and the clinical utility of Mp1p testing in asymptomatic people with AHD, as well as in IRIS and relapsed disease.

## CRediT authorship contribution statement

**Emily E. Evans:** Writing – review & editing, Writing – original draft, Visualization, Conceptualization. **Jennifer Spicer:** Writing – review & editing, Supervision, Investigation, Conceptualization. **Vince Marconi:** Writing – review & editing, Supervision. **Thuy Le:** Writing – review & editing, Visualization, Supervision, Investigation, Conceptualization.

## Ethical Form

Please note that this journal requires full disclosure of all sources of funding and potential conflicts of interest. The journal also requires a declaration that the author(s) have obtained written and signed consent to publish the case report from the patient or legal guardian(s).

The statements on funding, conflict of interest and consent need to be submitted via our Ethical Form that can be downloaded from the submission site www.ees.elsevier.com/mmcr. **Please note that your manuscript will not be considered for publication until the signed Ethical Form has been received.**

## Conflict of interest

Dr. Thuy Le receives investigator-initiated research grant from Gilead Sciences. VCM has received investigator-initiated research grants (to the institution) and research support from Eli Lilly, Bayer, Gilead Sciences, Merck, Pfizer, and ViiV.
